# The Relationship Between Decisional Regret and Well-Being in Patients With and Without Depressive Disorders: Mediating Role of Shared Decision-Making

**DOI:** 10.3389/fpsyt.2021.657224

**Published:** 2021-06-16

**Authors:** Richard Huan Xu, Ling-ming Zhou, Dong Wang

**Affiliations:** ^1^Department of Rehabilitation Sciences, The Hong Kong Polytechnic University, Hong Kong, China; ^2^Center for Health Systems and Policy Research, Jockey Club School of Public Health and Primary Care, The Chinese University of Hong Kong, Hong Kong, China; ^3^School of Health Management, Southern Medical University, Guangzhou, China

**Keywords:** decisional regret, subjective well-being, depression, shared decision-making, mediation analysis

## Abstract

**Background:** The objectives of this study were two-fold: (1) to assess the relationship between patients' decisional regret and their well-being and (2) to examine the mediated effect of shared decision-making (SDM) on this relationship.

**Methods:** A cross-sectional survey was conducted in five cities in Southern China. Patients were asked to fill out questionnaires assessing their decisional regret, SDM, subjective well-being, and depressive status. Mediation analysis was used to investigate the effect of SDM on the relationship between patients' decisional regret and their subjective well-being.

**Results:** The findings showed significant direct negative effects of decisional regret on subjective well-being and SDM. For non-depressive patients, SDM exerted a significant and indirect effect on reducing the negative influence of decisional regret on subjective well-being.

**Conclusions:** Findings suggest that implementation of SDM can decrease patients' decisional regret and improve their well-being; however, there is a need to examine their depressive status as part of routine healthcare.

## Introduction

While sophisticated medical technologies are already available in a growing number of healthcare services, given that the risks and benefits of a treatment cannot be directly predicted due to each patient's individual characteristics, most medical decisions must be made in a context of uncertainty (1). Several studies have indicated that, sometimes, even patients' preferences, and needs have been considered in the treatment; if adverse or unfavorable outcomes occur, these unexpected results are likely to generate negative emotions, such as regret, in or after the treatment (2, 3).

Regret in medical care often refers to the fact that some aspects of physical or mental health have not been regained as the interventions intended (4). If a patient obtains an undesirable result after treatment, he/she tends to regret the decision-making process (5). In order to improve the quality of healthcare, clinical professionals currently prefer to provide the “standard of care” for patients, which is the treatment that a multidisciplinary team or guidelines recommend. However, the standard of care might be not the “right” care for individual patients, as it usually does not consider the patient's personality, physical or psychological characteristics, life experiences, and socioeconomic status (SES) (6). There is growing recognition that an objectively appropriate treatment can, sometimes, lead to negative emotions and poor health among patients. Wilson et al. found that self-reported decisional regret was present in ~1 in seven surgical patients (7). Several studies have demonstrated a relationship between high decisional regret and low levels of well-being. For example, Kvale et al. found that unfulfilled goals are associated with depression (8). Lecci et al. ascertained that decisional regret results in low levels of life satisfaction (9).

Considerable research has indicated that involving patients in the shared decision-making (SDM) process is an effective way to develop a trustful patient–doctor relationship, reduce negative emotions, and increase patients' well-being (10, 11). SDM could help patients recognize multiple possible outcomes by communicating with healthcare providers and developing realistic expectations about the possible outcomes of the treatment (12). Jokisaari indicated that patients' perception of their physical and mental health after treatment affected their satisfaction with pretreatment decision-making (13). Hong et al. also demonstrated that parents of children who received otoplasty perceived themselves as being more involved in the decision-making process reported less decisional conflict and decisional regret (14). In addition, the relationship between SDM and increased life satisfaction, quality of life (QoL), and well-being has also been documented. For example, Hughes et al. indicated that SDM was associated with satisfactory patient-reported health outcomes (15). Taylor et al. also demonstrated that the implementation of SDM is associated with improved QoL and disease control (16). However, the general idea was that regret in healthcare is less likely to be avoided, even if a better decision was made in the treatment. McQueen indicated that some regrets are not due to flaws in decision making but are unpredicted and uncontrollable from the patients' perspective when they made the decision (17). SDM aims to help clinical professionals and patients share the same orientation and enhance the quality of communication and patient satisfaction (18). It is crucial to provide patients with sufficient information and then help them to make a better decision that fulfills their preferences and needs in complex healthcare. However, currently, there is a dearth of evidence identifying and quantifying the effect of SDM on the relationship between patient regret and well-being. To address this issue, we hypothesized that decisional regret is likely to have both a direct and indirect influence on subjective well-being through SDM.

## Methods

### Data Source and Collection

The data used in this study were obtained from a multicentered cross-sectional survey that investigated patient-centered care (PCC) in public hospitals in China from November 2019 to January 2020. Patients were recruited from eight hospitals in five cities (Guangzhou, Shenzhen, Zhanjiang, Meizhou, and Shaoguan) of Guangdong Province. All patients from the target hospitals were invited to participate in the study during the survey period. The inclusion criteria were (a) ≥18 years, (b) can read and speak Chinese, (c) has no cognitive problems, and (d) able to complete the informed consent form. With the assistance of ward nurses, all eligible patients were asked to complete a structured questionnaire during a face-to-face interview, which gathered information about their demographic characteristics, SES, health conditions, well-being, lifestyle, use of health services, and attitudes toward PCC. The data of 704 patients who met the inclusion criteria and successfully completed the targeted measurements were analyzed.

### Measurement

The measures included four sections: patients' characteristics, SDM, subjective well-being, and decisional regret.

#### Patient Characteristics

Patient demographic and socioeconomic characteristics, including gender, age, educational level, family register, living and working status, chronic conditions, body mass index, and disease severity, were collected.

#### Shared Decision-Making

The CollaboRATE scale is a questionnaire that measures SDM (19). It has three items and assesses SDM from different aspects [1. medical professionals help patients to understand their health issues (UND); 2. medical professionals communicate the most important health issues with patients (COM); and 3. medical professionals consider patients' preference in their healthcare plan (PRE)]. The Chinese version of CollaboRATE contains a scale that ranges from 0 to 10 for each item, where 0 represents “no effort was made” and 10 represents “every effort was made” by the medical professional to promote SDM. The psychometric properties of the CollaboRATE have been reported elsewhere (20), but not in a Chinese population. In this study, Cronbach's alpha was 0.93, which indicated a satisfactory internal consistency reliability.

#### Subjective Well-Being

ICEpop CAPability measure for Adults (ICECAP-A) is a generic and preference-based instrument that evaluates an individual's well-being (21). The descriptive system of the ICECAP-A has five items (stability, attachment, autonomy, achievement, and enjoyment), and each item has four response options that range from fully capable to not capable. Currently, no value set exists for the Chinese population; therefore, in order to calculate one's well-being, we calculated the sum of the five items. We reversed and recoded the sum score of the ICECAP-A that a high score indicates good well-being. The psychometric properties of the Chinese ICECAP-A have been reported by Tang et al. (22). In this study, Cronbach's alpha was 0.81, which indicated a satisfactory internal consistency reliability.

#### Decisional Regret

Decisional regret scale (DRS) is a five-item unidimensional self-report scale that assesses patients' decisional regret (10). It uses a five-point Likert scale ranging from 1 (“strongly agree”) to 5 (“strongly disagree”). Items 2 and 4 are reverse scored. The overall score is transformed from 0 to 100 by subtracting 1 from each item and then multiplying by 25. A lower overall score indicates few regrets, whereas a higher overall score indicates more regrets. The psychometric properties of the Chinese DRS have been reported by Xu et al. (23). In this study, Cronbach's alpha was 0.74, which indicated an acceptable internal consistency reliability.

#### Depressive status

Patient Health Questionnaire-2 (PHQ-2) was used to measure the patients' depressive status. The PHQ-2 includes the first two items of the PHQ-9 (24), which is the depression module from the full PHQ. The patients were asked to recall the frequency of a depressed mood and anhedonia over the past 2 weeks. A PHQ-2 score ≥3 (0–6) is considered to indicate a depressed mood. The psychometric properties of the Chinese version of the PHQ-2 have been reported by Liu et al. (25). In this study, Cronbach's alpha was 0.78, which indicated an acceptable internal consistency reliability.

### Ethical Approval

The study protocol and informed consent were approved by the Institutional Review Board of the Second Affiliated Hospital of Guangzhou Medical University (ID: 2019-ks-28).

### Statistical Analysis

Descriptive analysis was used to describe the patients' characteristics. The relationships among all the study variables were examined using Spearman's rank correlation coefficient (ρ), where ρ > 0.3 indicates a moderate correlation (26). Structural equation modeling (SEM) was used to examine the direct and indirect effects of decisional regret on patients' well-being and mediated by SDM. In order to alleviate the potential effect of depressive status (confounder) on the regret–SDM–well-being relationship, we divided all the patients into two subgroups (depression and non-depression group) based on the results of PHQ-2 (depression group: PHQ-2 ≥ 3). Considering that some data were not normally distributed, the maximum likelihood estimation (MLR) with robust standard errors that are robust to non-normality and non-independence of observations was used to estimate the model. The estimates of the indirect effects were based on running 500 bootstrap iterations of computed samples at 95% confidence intervals (95% CIs). Overall, eight models were developed to estimate the relationship between SDM, decisional regret, and well-being. The effect of SDM was estimated from the perspective of both scale level (CollaboRATE overall score) and item level (scores of UND, COM, and PRE). The first four models (Models 1–4) used the subsample of patients with depression, and the second four models (Models 5–8) used the subsample of patients without depression. For both depressive and non-depressive subsamples, the first model was CollaboRATE overall score model (Model 1 and Model 5), the second model was UND score model (Model 2 and Model 4), the third model was COM score model (Model 3 and Model 6), and the fourth model was PRE score model (Model 4 and Model 8). Multivariable regression analysis was performed to further investigate the association of patients' well-being with decisional regret and its interaction with SDM adjusted by their background characteristics. R software (R Foundation, Vienna, Austria) was used for the data analysis (mediation analysis was conducted by using package “*lavaan”*). The *p*-value was set at ≤ 0.05.

## Results

### Patient Characteristics

Participants sampled for the study had a mean age of 49.3 years [standard deviation (SD) = 17.3], and more than half of them were women. The majority of the participants had completed secondary or higher education (80.1%), and 63.8% of their families were registered in the urban area. Moreover, ~60% of respondents were fully employed, 53% self-reported having some chronic conditions, and 73.6% reported living with a life-threatening condition, which in 19.2, 34.2, and 30.2% of cases was minor, moderate, and severe, respectively. In terms of depressive disorder, 46.3% of the patients were suffering from depression to some extent (Table 1).

**Table 1:** The demographics of patients (*n* = 702).

	**Value**
**Sex**, ***n*** **(%)**	
Male	337 (48.0)
Age (years), mean (SD)	49.3 (17.3)
**Educational level**, ***n*** **(%)**	
No/primary	139 (19.9)
Secondary/post-secondary	427 (61.2)
Tertiary or above	132 (18.9)
**Family register**, ***n*** **(%)**	
Urban area	438 (63.8)
Rural area	249 (36.2)
**Living status**, ***n*** **(%)**	
Live lone	43 (6.2)
Live with families	644 (92.5)
**Working status**, ***n*** **(%)**	
Fully employed	399 (56.8)
Non-employed	89 (12.7)
Retired	214 (30.5)
**Chronic condition**, ***n*** **(%)**	
No	323 (47.0)
Yes	364 (53.0)
**BMI**, ***n*** **(%)**	
≤ 18.4	67 (9.7)
18.5–22.9	312 (45.0)
≥23	314 (45.3)
**Severity of disease**, ***n*** **(%)**	
No threat to life	111 (16.4)
Minor threat to life	130 (19.2)
Moderate threat to life	232 (34.2)
Severe threat to life	205 (30.2)
**Depressive status**	
Yes	297 (46.3)
No	345 (53.7)

### Correlations Between Measures

Table 2 shows that regret significantly correlated with SDM (*r* = −0.35) and well-being (*r* = −0.23). In terms of depressive disorder, the relationship with SDM (*r* = −0.36) and well-being (*r* = −0.22) was weaker among patients without depressive disorder than it was among patients with depressive disorder. Moreover, SDM was significantly and positively related to well-being (*r* = 0.19 and *r* = 0.32) in patients with either depressive or non-depressive disorders, which suggests that better SDM leads to higher well-being. The correlations between three domains of SDM with the well-being and decisional regret could be identified in Table 2.

**Table 2:** Descriptive statistics and correlation between measures.

	**Mean**	**SD**	**Range**	**Correlation coefficient (95% CI)**		
				**REG**	**WEL**	**SDM**
**Overall**						
REG	23.81	16.25	0–70	–	–	–
SDM (Overall)	24.58	3.3	9–27	−0.35 (−0.41, −0.28)	–	–
SDM (UND)	8.21	1.14	3–9	−0.33 (−0.4, −0.26)	0.25 (0.18, 0.32)	–
SDM (COM)	8.22	1.11	3–9	−0.33 (−0.4, −0.26)	0.3 (0.22, 0.36)	–
SDM (PRE)	8.15	1.26	1–9	−0.33 (−0.39, −0.26)	0.28 (0.21, 0.35)	–
WEL	15.72	2.57	7–20	−0.23 (−0.3, −0.16)	–	0.29 (0.22, 0.36)
**Depressive status**						
REG	26.67	14.55	0–70	–	–	–
SDM (Overall)	23.98	3.39	9–27	−0.33 (−0.43, −0.22)	–	–
SDM (UND)	8.02	1.18	3–9	−0.3 (−0.4, −0.19)	0.16 (0.05, 0.28)	–
SDM (COM)	8.0	1.15	3–9	−0.32 (−0.43, −0.22)	0.21 (0.1, 0.32)	–
SDM (PRE)	7.95	1.3	1–9	−0.29 (−0.39, −0.18)	0.16 (0.05, 0.27)	–
WEL	14.64	2.17	7–20	−0.16 (−0.27, −0.05)	–	0.19 (0.07, 0.29)
**Non-depressive status**						
REG	21.83	16.99	0–60	–	–	–
SDM (Overall)	24.96	3.25	12–27	−0.36 (−0.45, −0.27)	–	–
SDM (UND)	8.31	1.14	3–9	−0.36 (−0.45, −0.26)	0.28 (0.18, 0.38)	–
SDM (COM)	8.37	1.07	4–9	−0.34 (−0.43, −0.24)	0.29 (0.19, 0.39)	–
SDM (PRE)	8.28	1.2	3–9	−0.36 (−0.44, −0.27)	0.34 (0.24, 0.44)	–
WEL	16.66	2.53	10–20	−0.22 (−0.32, −0.11)	–	0.32 (0.22, 0.41)

*For REG, a higher score means more regret; for SDM, a higher score means better collaboration; for WEL, a higher score means better well-being*.

### Testing for Effects (Mediation Analysis)

For patients with depressive disorder, an effect analysis showed that patients' well-being was significantly affected by decisional regret (*b* = −0.233, *z* = −2.572, *p* = 0.01) and SDM (*b* = 0.135, *z* = 4.963, *p* < 0.001), respectively. That is, higher levels of well-being were associated with more regret, but better SDM. The analysis of an indirect effect showed that regret indirectly affects well-being through SDM (*b* = −0.031); however, the relationship was statistically non-significant, and the bootstrapped 95% CI for the indirect effect included zero (−0.063, 0.001). While examining the mediated effect of SDM subdomains on regret-well-being relationship, we found that only communication significantly reduce the negative effect of regret on the patients' well-being (*b* = −0.033, *z* = −2.076, *p* = 0.038).

**Figure 1:**
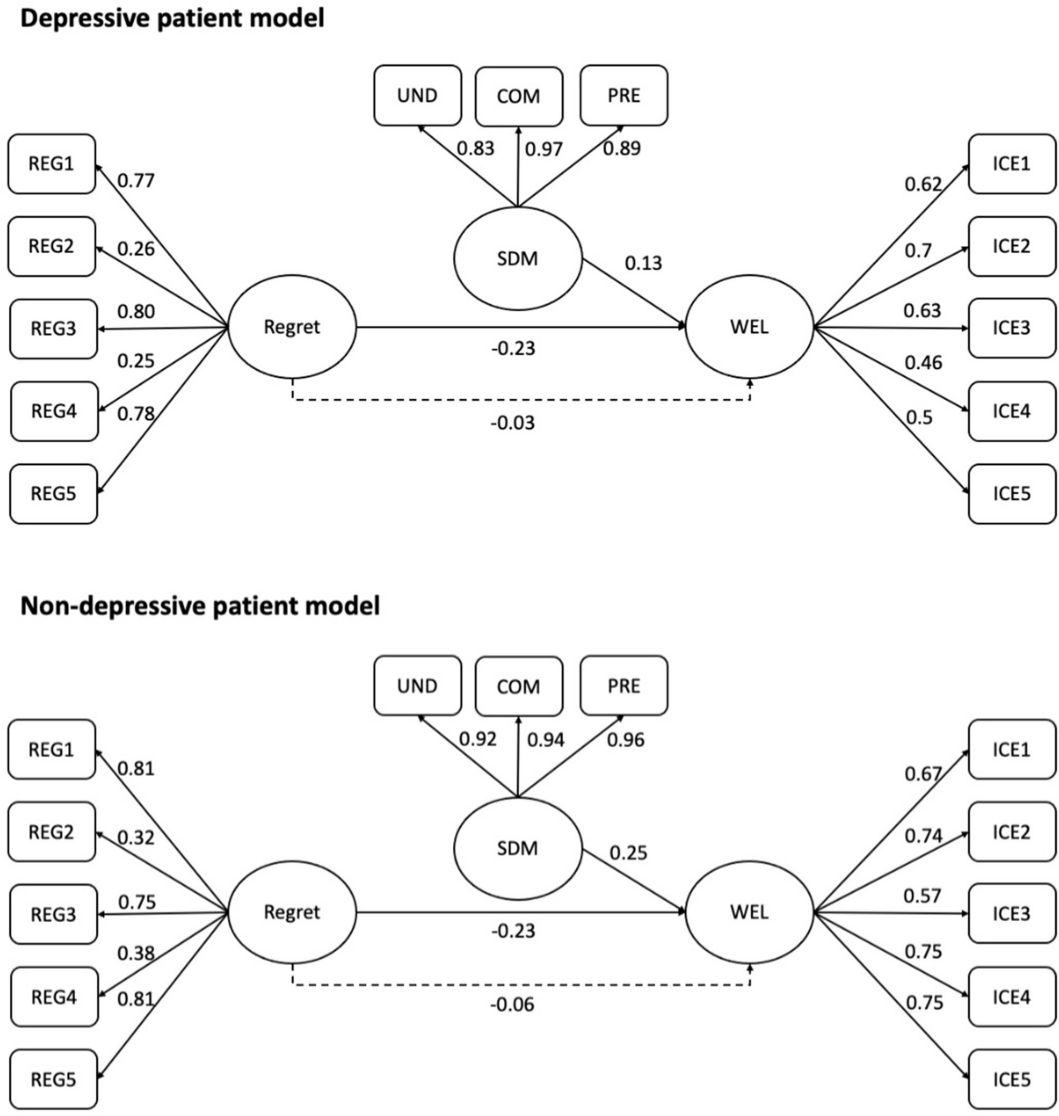
The observed path models for patients with/without depression.

For patients without depressive disorder, the pattern was similar in that high levels of well-being were related to low levels of regret (*b* = −0.515, *z* = −7.616, *p* < 0.001) but improved SDM (*b* = 0.247, *z* = 3.258, *p* = 0.001). However, compared with patients with depressive disorder, patients without depressive disorder demonstrated SDM results in a higher level of well-being (0.135 vs. 0.247). An analysis of the indirect effect showed that regret significantly affects well-being through SDM (*b* = −0.057, *p* < 0.001); the bootstrapped 95% CI for the indirect effect was entirely below zero (−0.087, −0.027). For subdomain analysis, we found that the negative effects of regret on patients' well-being were significantly reduced by all the three subdomains of SDM (Table 3). Figure 1 presents the observed path models for patients with/without depression.

**Table 3:** Direct and indirect effects and 95% confidence intervals for the models.

**Model pathway**	**Coefficient**	**SE**	***z*-value**	***p*-value**	**95% CI**	
					**Lower**	**Higher**
**Model 1**						
Direct effect						
REG → WEL	20.233	0.091	22.572	0.01	20.411	20.055
SDM → WEL	0.135	0.083	4.963	<0.001	0.24	0.552
Indirect effect						
REG → SDM → WEL	20.031	0.016	21.916	0.05	20.063	0.001
**Model 2**						
Direct effect						
REG → WEL	20.262	0.087	23.03	0.002	20.432	20.093
UND → WEL	0.08	0.078	1.02	0.31	20.073	0.233
Indirect effect						
REG → UND → WEL	20.021	0.018	21.134	0.257	20.057	0.015
**Model 3**						
Direct effect						
REG → WEL	20.232	0.089	22.612	0.009	20.405	20.058
COM → WEL	0.143	0.078	1.823	0.06	20.011	0.296
Indirect effect						
REG → COM → WEL	20.033	0.016	22.076	0.038	20.064	20.002
**Model 4**						
Direct effect						
REG → WEL	20.261	0.089	22.933	0.003	20.436	20.087
PRE → WEL	0.078	0.095	0.83	0.407	20.107	0.264
Indirect effect						
REG → PRE → WEL	20.021	0.02	20.93	0.352	20.064	0.023
**Mode 5**						
Direct effect						
REG → WEL	20.23	0.086	22.695	0.007	20.398	20.063
SDM → WEL	0.247	0.076	3.258	0.001	0.098	0.396
Indirect effect						
REG → SDM → WEL	20.057	0.015	23.733	<0.001	20.087	20.027
**Model 6**						
Direct effect						
REG → WEL	20.262	0.085	23.066	0.002	20.429	20.094
UND → WEL	0.199	0.072	2.769	0.006	0.058	0.34
Indirect effect						
REG → UND → WEL	20.052	0.014	23.757	<0.001	20.079	20.025
**Model 7**						
Direct effect						
REG → WEL	20.256	0.081	23.156	0.002	20.415	20.097
COM → WEL	0.217	0.075	2.888	0.004	0.07	0.364
Indirect effect						
REG → COM → WEL	20.055	0.016	23.579	<0.001	20.086	20.025
**Model 8**						
Direct effect						
REG → WEL	20.224	0.082	22.727	0.006	20.386	20.063
PRE → WEL	0.279	0.068	4.091	<0.001	0.145	0.413
Indirect effect						
REG → PRE → WEL	20.063	0.016	23.917	<0.001	20.094	20.031

*SE, standard error; 95% CI, 95% confidence interval; REG, regret; SDM, shared decision-making; WEL, well-being*.

Results of multivariable regression analysis indicated that, after adjusted by patients' SES and chronic condition status, there is a negative relationship between patients' well-being and their level of decisional regret (*b* = −0.092, *p* = 0.04). The interaction between decisional regret and SDM reduces the negative effect of decisional regret on patients' well-being (*b* = −0.004, *p* = 0.022) (Table 4).

**Table 4:** Results of regression analysis.

	***b***	**Std. error**	***p*-value**
Decisional regret	−0.092	0.046	0.04
SDM	0.276	0.064	<0.001
Decisional regret * SDM	−0.004	0.002	0.022
Depressive disorder	−0.637	0.061	<0.001
Female	−0.101	0.19	0.596
Age	−0.021	0.008	0.01
Secondary/post-secondary	0.026	0.256	0.919
Tertiary or above	−0.197	0.357	0.582
Rural resident	−0.621	0.226	0.006
Live with family	0.327	0.33	0.322
Non-employed	−0.42	0.303	0.167
Retired	−0.032	0.279	0.91
No chronic condition	0.007	0.211	0.973

*Reference: male, no/primary, urban resident, live alone, fully employed and with chronic conditions*.

*B, coefficient; Std. Error, standard error*.

## Discussion

### Principal Findings

This is the first study of its kind to quantify the relationship between decisional regret and subjective well-being mediated by SDM in a large sample of Chinese patients. The results showed that, among them, high levels of decisional regret significantly correlated with low levels of subjective well-being. However, SDM exerted a positive mediating effect that alleviated the negative influence of decisional regret on subjective well-being. That is, patients who were involved in SDM were more likely to report a lower level of impairment on their well-being than the patients who were not involved in the SDM, insofar as they reported living without depressive disorder. This study presents initial empirical evidence that SDM could be a vital way that not only directly improves patients' well-being but also indirectly alleviates the influence of negative emotions on their well-being. Thus, the findings may provide researchers with insight into the application of SDM for individuals across health contexts. Providing training in SDM for healthcare professionals, especially, improve their skills to listen from patients' perspective and communicate with them, which could be helpful in tailoring health information, explaining health-related behaviors, and enhancing patients' well-being and QoL.

### Comparisons With Previous Studies

No study has directly assessed the relationship between patients' decisional regret and well-being mediated by SDM in neither Western nor Asian countries. Some previous studies have discussed the correlation between decisional regret and SDM. For example, Hong et al. demonstrated that parents of children who participated in consultation for otoplasty surgery reported lower decisional conflict and regret when they perceived themselves as being more involved in the decision-making (14). Davidson and Goldenberg indicated that, in their study, 63/67 patients with prostate cancer participated in medical decision-making (27). Although several studies have examined the topic of regret, most reports involved a limited number of patients [e.g., sample size is 65 in a study by Hong et al. (14)]. Hickman et al. suggested that researchers need to evaluate the effect of decisional conflict or regret in a larger and more diverse sample (28). As observed in our large sample, SDM is important in enhancing the engagement of stakeholders, which is a valid strategy to help and encourage patients to think more elaborately before making a choice and reducing the after-decisional negative emotions. Nevertheless, Wilson et al. indicated that the role of SDM might vary in reducing postoperative regret in different disease processes (7).

Studies investigating the relationship between SDM and subjective well-being are limited. A randomized controlled trial (study) in the Netherlands indicated that, in the long term, patients in the SDM group showed less intrusive thoughts better general health and tended to be less depressed (29). A systematic review examining the relationship between SDM and QoL demonstrated a positive relationship between SDM and QoL; however, few studies have directly assessed the concept of SDM but have largely evaluated other similar concepts, such as patient involvement, which might not capture the full picture of SDM (30). Another systematic review identified SDM as an effective way to improve patient experience, such as satisfaction, treatment adherence, and their health status, rather than well-being. Additionally, the evidence for the effectiveness of SDM in improving patients' long-term care is inconclusive (31). Compared with QoL, well-being tends to be used in a more psychologically or spiritually oriented perspective (32). It seems to be a more reasonable indicator than the others to assess the impact of negative emotions during treatment.

In this study, compared with non-depressive patients, among patients with depressive disorder, regret had a stronger correlation with SDM but a weaker correlation with wellbeing. Several studies have reported that patients receiving mental care are highly likely to report receiving merits from the SDM (33, 34). Swanson et al. further found that SDM and receipt of mental healthcare are positively associated with patient satisfaction (35). However, in this study, the finding that the negative influence of regret on well-being could be significantly alleviated through SDM held true only for patients without depressive disorder. Limited studies have investigated the relationship between depression, regret, and SDM. Wilson et al. found that patients experiencing regret were more likely than patients not experiencing regret to exhibit depression (36). Thus, in this study, the finding that depressive patients reported low well-being might be because they experienced more regret than non-depressive patients in the healthcare sector. Additionally, Drake indicated that the integrative and person-centered healthcare system tends to provide effective mental healthcare (17); however, currently in China, the healthcare system is fragmented, and the doctor- or treatment-centered pattern is still dominant (37). Thus, SDM among patients with unsatisfied mental care may undermine the positive effect of SDM on the relationship between regret and well-being among patients in China.

Further, we found that patients who are old and living in rural areas are more likely to report a lower well-being than their counterparts. It was consistent with some findings reported by previous studies that age-related differences in patient well-being suggest a role for age-sensitive interventions in the treatment (38). In the context of the urban–rural dual system in China, the health inequity, e.g., lack of drugs and quality healthcare professionals in rural areas, is ubiquitous (39). Our findings extended the knowledge about rural residents' health related well-being in China; however, in this study, all participants were recruited from urban hospitals, despite that half of them confirmed they are rural residents; based on our experience, most of them were living in the city but hold a rural resident registry certificate. Thus, further studies are needed to investigate the relationship between decisional regret and wellbeing in patients living in rural areas.

### Strengths and Limitations

This study has several strengths. First, this was the first study to assess and quantify the relationship between patients' decisional regret, SDM, and well-being in China. The findings confirmed that SDM is a useful tool to diminish the effect of a patient's negative emotions in healthcare. Second, we used the preference-based ICECAP-A, based on extra-welfarism theories, to evaluate patients' capability well-being. The results might be important to help decision makers consider the role of patients' emotions (non-economic factors) in comparing the cost-effectiveness of health and social interventions and policies.

The limitations of this study should be addressed. First, this was a cross-sectional study; thus, no causal relationship could be established from the analysis. Longitudinal data should be collected and presented in future studies. Second, we did not examine the relationships between regret, wellbeing, and SDM among patients with different diseases. Given the various clinical characteristics and treatment preferences for different patient groups, it may weaken the generalizability of our findings to some extent. Third, the psychometric properties of the DRS (a paper that assesses the validity of the Chinese DRS is under review) and CollaboRATE were not confirmed in the Chinese population, which might have resulted in some uncertainties in interpreting our findings.

## Conclusion

This study provides empirical evidence that there is a relationship between increased decisional regret and decreased subjective well-being among patients in China. Through SDM, the negative impact of decisional regret on well-being was alleviated. However, the presence of depressive disorder undermined the effects of SDM on alleviating the negative influence of regret on patients' well-being. Further, there is a need to conduct a longitudinal study to examine the well-being trajectory of the patients and how it can be affected by SDM and decisional regret. Furthermore, studies examining decisional aid to assist patients in choosing among different medical treatment options should be conducted in the future.

## Data Availability Statement

Raw data supporting the findings of this study are available from the corresponding author on request.

## Ethics Statement

The studies involving human participants were reviewed and approved by the Institutional Review Board of the Second Affiliated Hospital of Guangzhou Medical University. The patients/participants provided their written informed consent to participate in this study.

## Author Contributions

RX and DW: conceptualization. RX: formal analysis, software, and writing—original draft. L-mZ: investigation. RX and L-mZ: methodology. DW: project administration. L-mZ and DW: resources. RX, L-mZ, and DW: writing—review and editing. All authors contributed to the article and approved the submitted version.

## Conflict of Interest

The authors declare that the research was conducted in the absence of any commercial or financial relationships that could be construed as a potential conflict of interest.
